# Supplementary material for: Thioesterase superfamily member 1 undergoes stimulus-coupled conformational reorganization to regulate metabolism in mice
Source: Nat Commun. 2021 Jun 9;12:3493. doi: 10.1038/s41467-021-23595-x (PMC8190112; doi:10.1038/s41467-021-23595-x)
Supplement: Supplementary file 1 — Supplementary Information [file 41467_2021_23595_MOESM1_ESM.pdf]

```
                          15 18      25
Human             1MIQNVGNHLRRGLASVFSNRTSRKSALRAG--N-D-SAMADGEG40
Gorilla           1MIQNVGNHLRRGLASVFSNRTSRKSALRAG--N-D-SAMADGEG40
Flying Lemur      1MIQNVGSHLRRGFASVFSSRTSRKSASRAG--D-NDFAMAEGEG41
Wolf              1MIQNVGNHLRRGIASVFSSRTSRKSASRAE--K-DSGAMADGEG41
Sea Lion          1MIQNVGNHLRRGIASVFSSRTSRKSASRSE--H-ADGAMADGEG41
Fruit Bat         1MIQNVGNHLRRSFASMFSSRQSRKSTSRAE--D-D-GAMADGEG40
European Rabbit   1MIQTVGSHLRRGFASVFSSRTSRKSASRAG--D-ADGAMADGEG41
Camel             1MIQNVGNHLRRGLASVFSSRTSRKSALRAE--N-T---MAEGEG38
Alpaca            1MIQNVGNHLRRGLASVFSSRTSRKSASRAE--N-T---MAEGEG38
Minke Whale       1MIQNVGNHLRRGLASVFSNRASRKSASHTG--N-N---MAEGEG38
Baiji             1MIQNVGNHLRRGLASVFSNRASRKSASRTE--N-N--NMAEDEG37
Artic Squirrl     1MIQNVGNHLRRGLASVFSSRTSRKSVSRAG--D-DN-DMEEGEG40
Cattle            1MIQTVGNHLRRGLASVFSNRTSRKSASRTD--S-D--NMADGEG39
White Rhino       1MIQNVGTHLRRGLASVFSSRTSRKSASRAE--K-DGGVMAEGEG41
Meerkat           1MIQNVGNHLRRSIASVFSSRTSRKSASRAE--K-D-GAMADGEG40
Sheep             1MIQTVGSHLRRGLASVFSNRTSRKSASRTD--S-D--NMADGEG39
Cheetah           1MIQNVGNHLRRGFASVFSSRLSRKSASHAE--K-DDGAMAGGEG41
Goat              1MIQTVGSHLRRGLASVFSNRTSRKSASRTD--S-D--NMADGEG39
Beluga Whale      1MIQNVGNHLRRGLASVFSNRASRKSASRTE--N-N--NMAEDKG39
Wild Boar         1---------MQGLASVFSNRASKKSAPRSE--N----NMGDGEG26
Cat               1MIQNVGNHLRRGFTSVFSSRLSRKSASHAE--K-DDGAMAGGEG41
Leopard           1MIQNVGNHLRRGFASVFSSRMSRKSASHAE--K-DDGAMAGGEG41
Horse             1MIQNVGNHLRRGLSSVFSSRSARKSASRAD--K-DGGAMAAGEG41
Naked Mole Rat    1MIQNVGSHLRRSFASVFSNRTSRKSVSRAG--D-ED-TMANSEG40
Armadillo         1MIQNVGNHLRRGFTSVFSGRTSRKSASRARAED-ADGAMAD-EG42
Mouse             1MIQNVGNHLRRGFASMFSNRTSRKSISHPE--SGDPPTMAEGEG42
Wombat            1-MQNIGNQLRRGLTSIFSGRASRKQAAQGR--D-PAGTM---ET37
```

Supplementary Fig. 1 Conservation of the *Them1* mRNA sequence for amino acids S15, S18, and S25 at the N-terminus among human, mouse, and other species**.**

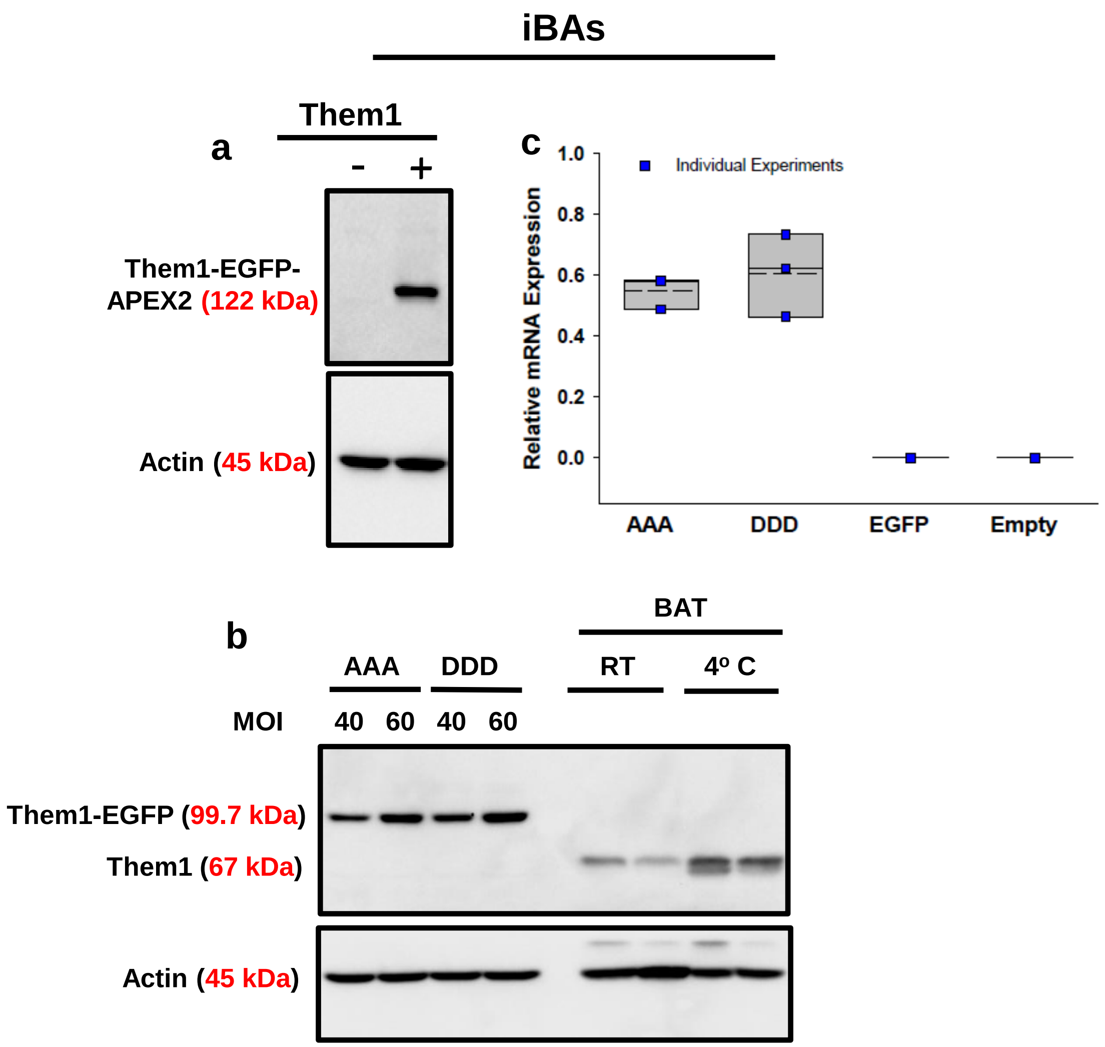

Supplementary Fig. 2 Expression levels of Them1 in immortalized brown adipose cells (iBAs) and in brown adipose tissue (BAT). **a** iBAs do not constitutively express Them1 protein. Transfection of a Them1-EGFP-APEX2 plasmid results in Them1 expression. Data are representative of n = 2 independent experiments. **b** Adenovirus (Ad) expressing the AAA- or DDD-Them1 mutant in iBAs at an MOI of 40 express Them1 to a similar level as BAT tissues from mice exposed to cold (4º C) for 48 h. Data are representative of n = 3 independent experiments. For BAT tissue, cold exposure for 48 h increases Them1 protein expression. Data are from 2 mice for room temperature (RT) and from 2 mice exposed to the cold (4º C) for 48 h. **c** Box and whisker plot to quantify the relative mRNA expression in iBAs transfected with Ad-AAA- or Ad-DDD-Them1 mutants compared to cells transfected with EGFP alone or cells transfected with an empty vector containing no Them1. For each box, a solid line represents the median and a dashed line is the mean. Outliers are shown at the 5th/95th percentiles. n=3 (3 replicates averaged from 3 individual experiments are shown in the blue squares). Immunoblot data were not processed or modified from the original scans.

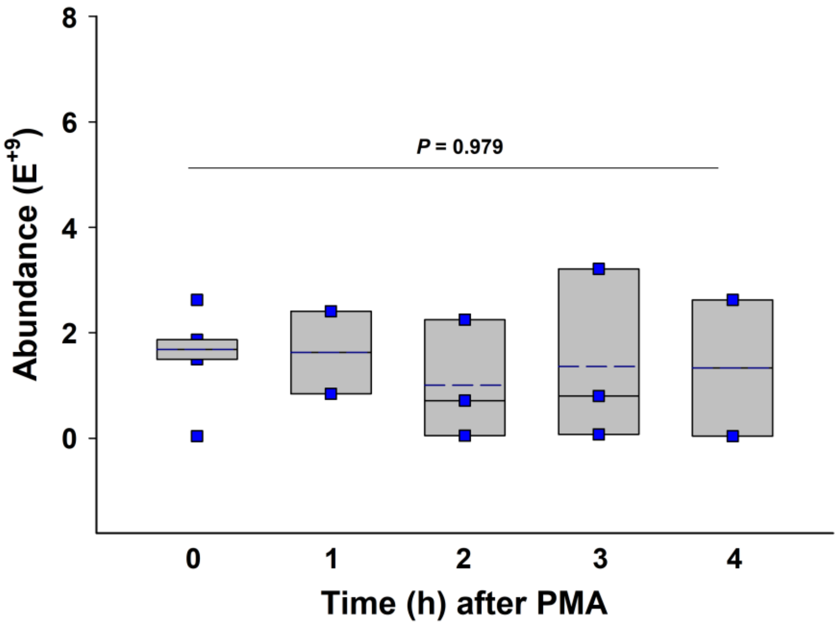

Supplementary Fig. 3 Isoform 2 of hormone-sensitive lipase (HSL, p54310), used as a housekeeping gene to normalize the LC-MS/MS data for Them1 (Fig. 1a), is not sensitive to stimulation with PMA. Box and whisker plot to quantify the abundance of phospho-peptides for HSL by LS-MS/MS after stimulation with PMA (3 μM). HSL, used to normalize the data across groups, did not change with time. For each box, a solid line represents the median and a dashed line is the mean. Outliers are shown at the $5^{th}/95^{th}$ percentiles. *n*=3 (3 replicates averaged from 3 individual experiments identified by the blue squares). *P* = 0.979 (no significant difference) was calculated from a one-way ANOVA.

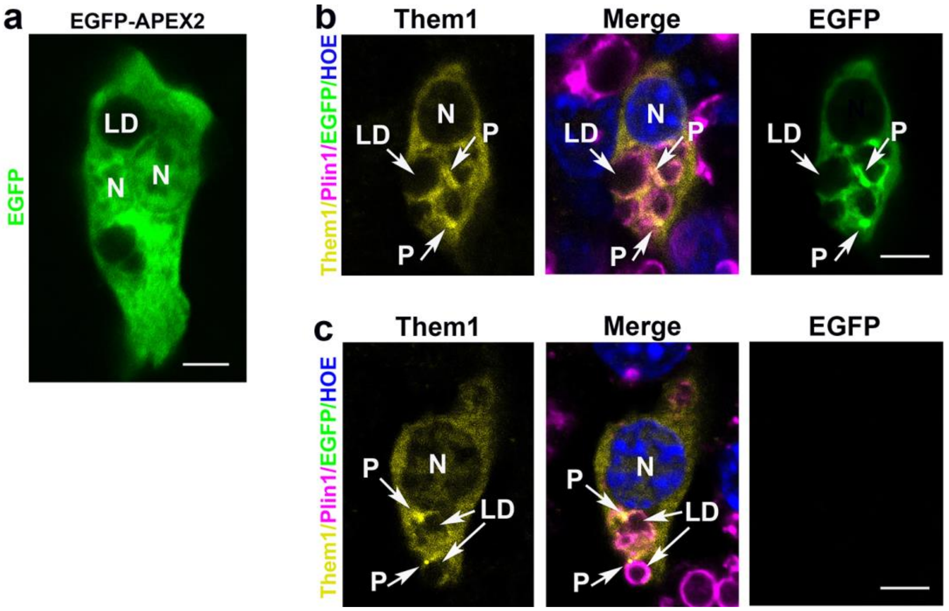

Supplementary Fig. 4 EGFP, per se, does not cause puncta formation in immortalized brown adipose cells (iBAs) transfected with Them1-EGFP-APEX2. **a** iBAs were transfected with a plasmid containing EGFP-APEX2 alone, without Them1. Note that the EGFP fluorescence signal is distributed uniformly throughout the cell cytoplasm and nucleus (N) excluding lipid droplets (LD). **b** iBAs were transfected with a plasmid containing Them1-EGFP-APEX2, fixed, and then stained with anti-Them1, anti-EGFP, anti-Plin1 to identify lipid droplets (LD) and Hoechst 33342 (HOE) to identify nuclei. Puncta (P), which were identified by immunofluorescence using anti-Them1 were in the same location as those positive for EGFP. **c** iBAs were transfected with a plasmid containing Them1 containing a stop codon (Them1-STOP) to prevent the translation of EGFP-APEX2. Them1, EGFP, Plin1, and HOE were evaluated by immunofluorescence, as above. Note that fluorescence signal for EGFP is not present in cells transfected with Them1-STOP and that Them1-containing puncta (P) are present in the cell cytoplasm, near lipid droplets (LD) when no EGFP is expressed. Data are representative of *n* = 3 cells from three different experiments with the same results. Scale bars, 10 μm.

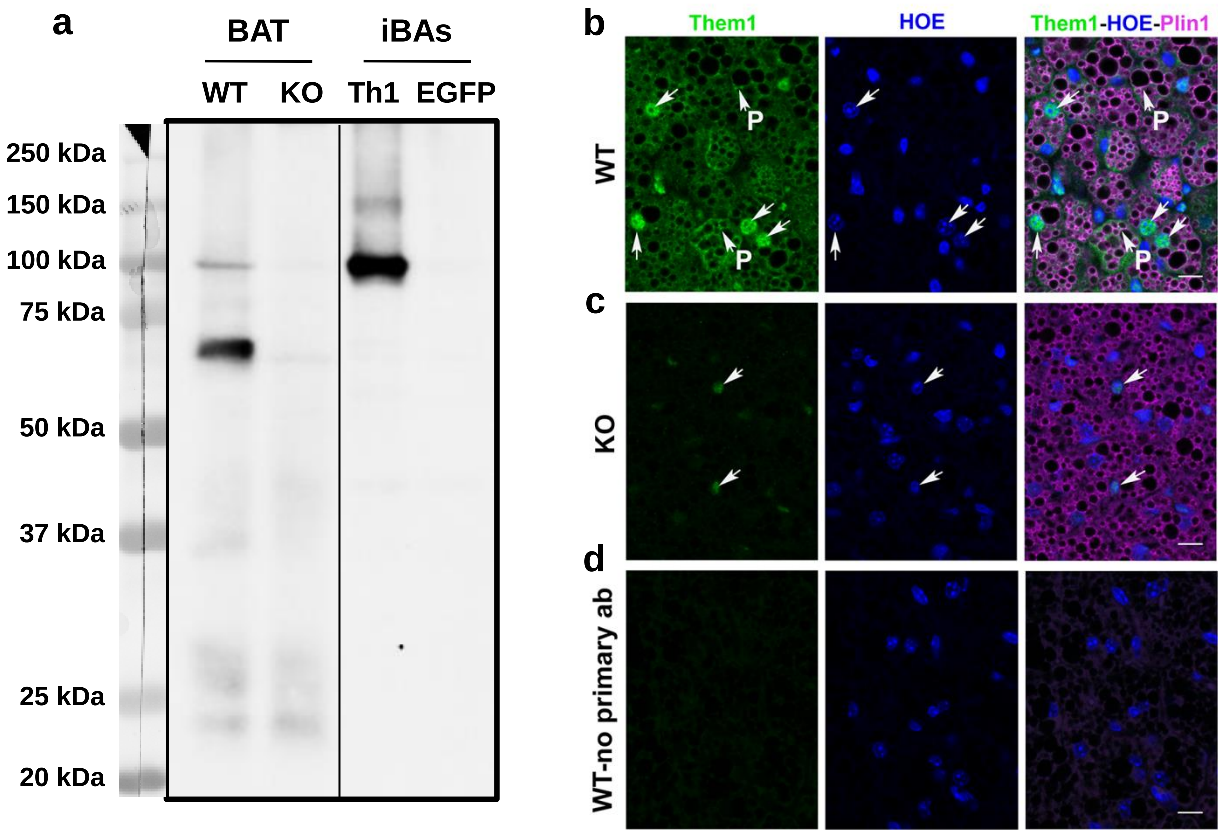

Supplementary Fig. 5 Characterization of the affinity purified rabbit anti-Them1 antibody used to identify Them1, Them1-EGFP-APEX2, and Ad-Them1-EGFP. The Them1 antibody used in this work was generated (by 21st Century Biologicals, Marlboro, MA) using a synthetic peptide to Them1 and then affinity purified using the same peptide. **a** Brown adipose tissue (BAT) from wild-type (WT) or Them1-deficient (KO) mice was homogenized and prepared for immunoblot analysis. Native Them1 is 67 kDa. These data are representative of two immunoblots that independently show the specificity of this antibody to Them1 in BAT. Additionally, immortalized brown adipose cells (iBAs) were transfected with Ad-Them1-EGFP (Th1) or Ad-EGFP and prepared for immunoblot analysis. Them1 linked to EGFP is 95 kDa. Note the specificity of this antibody to Them1 in transfected iBAs. **b-c** BAT from wild-type (WT) or Them1-deficient (KO) mice were stained with anti-Them1, Hoechst 33342 (HOE) to identify nuclei, and anti-perilipin (Plin1) to identify lipid droplets. **b** Them1 localized to puncta (P), was weakly diffuse in the cytoplasm, and also localized to some nuclei (arrows). **c** Them1 antibody showed weak background staining in some nuclei from BAT in KO mice. **d** Little background staining was observed in BAT from WT mice when deleting the primary antibody and using the secondary antibodies only. The images in b-d are representative of results from *n*=3 different dishes of cultured cells with the same results. Scale bars, 10 μm.

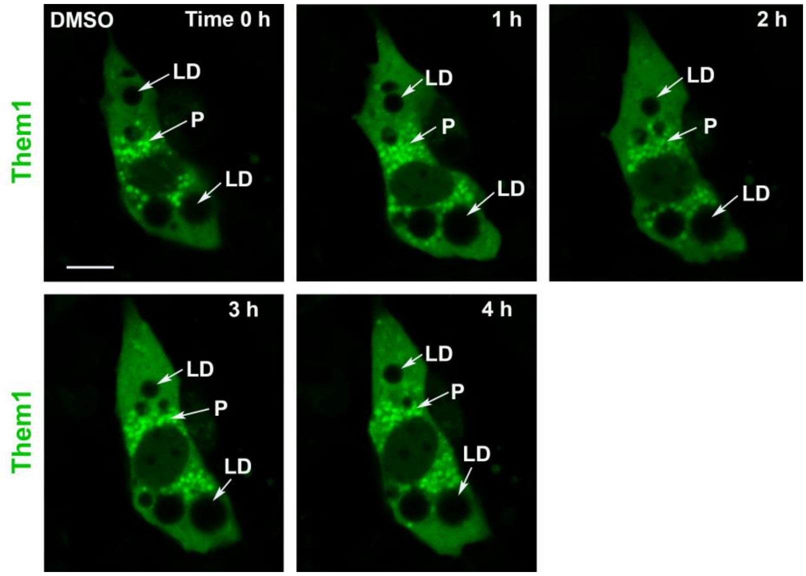

Supplementary Fig. 6 Neither DMSO nor laser light exposure affects puncta shape or intensity. Immortalized brown adipose cells expressing Them1 formed puncta that reorganize to a diffuse phenotype after the addition of PMA (see Fig. 1h).   This control experiment shows that there is no reorganization of puncta after the addition of 0.1% DMSO, which is the vehicle for PMA, and there is no reorganization of puncta shape or reduction of fluorescence signal intensity due to laser-induced damage after 4 h of data collection. Data are representative of n = 3 cells from 3 different experiments with the same results. LD, lipid droplets; P, puncta. Scale bar, 10 μm.

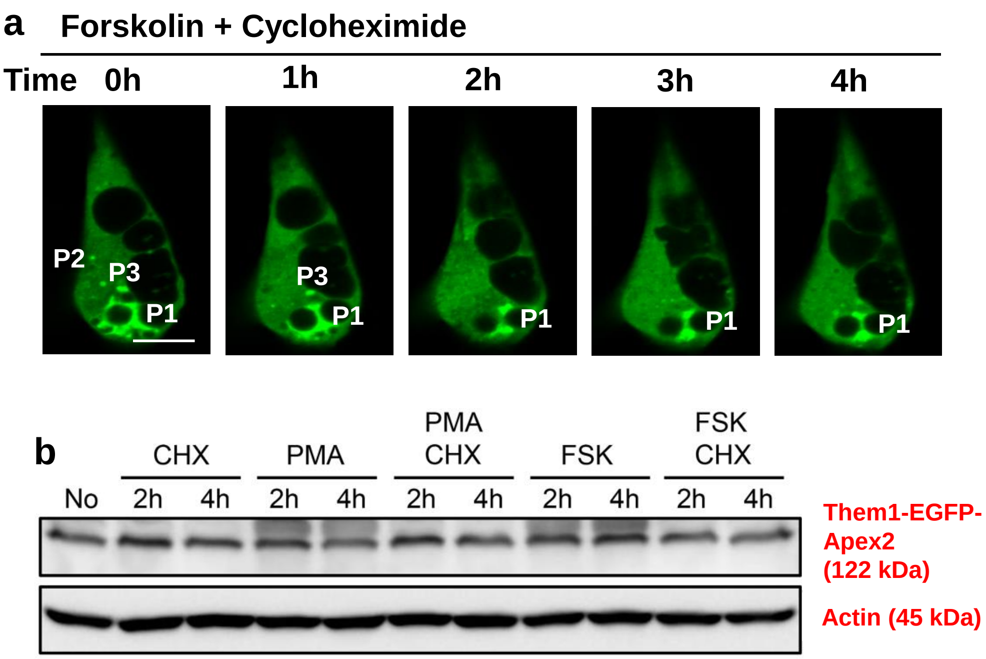

Supplementary Fig. 7 Blocking protein synthesis with cycloheximide has no effect on the diffusion of Them1 from puncta or the expression of intracellular Them1 in immortalized brown adipose cells (iBAs) after stimulation with forskolin or PMA. **a** Cycloheximide was added with forskolin to stimulate PKC activation and the dissolution of puncta (P). The dissolution of numerous puncta (P1, P2, and P3) were demonstrated over time in the presence of cycloheximide, suggesting that protein synthesis is not needed for the dissolution of puncta. Scale bar, 10 μm. **b** Immunoblot from iBAs treated with cycloheximide alone (CHX), PMA alone, PMA plus cycloheximide, forskolin (FSK), or forskolin plus cycloheximide. Data are representative of *n* = 3 independent experiments with the same results. Immunoblot data were not processed or modified from the original scans.

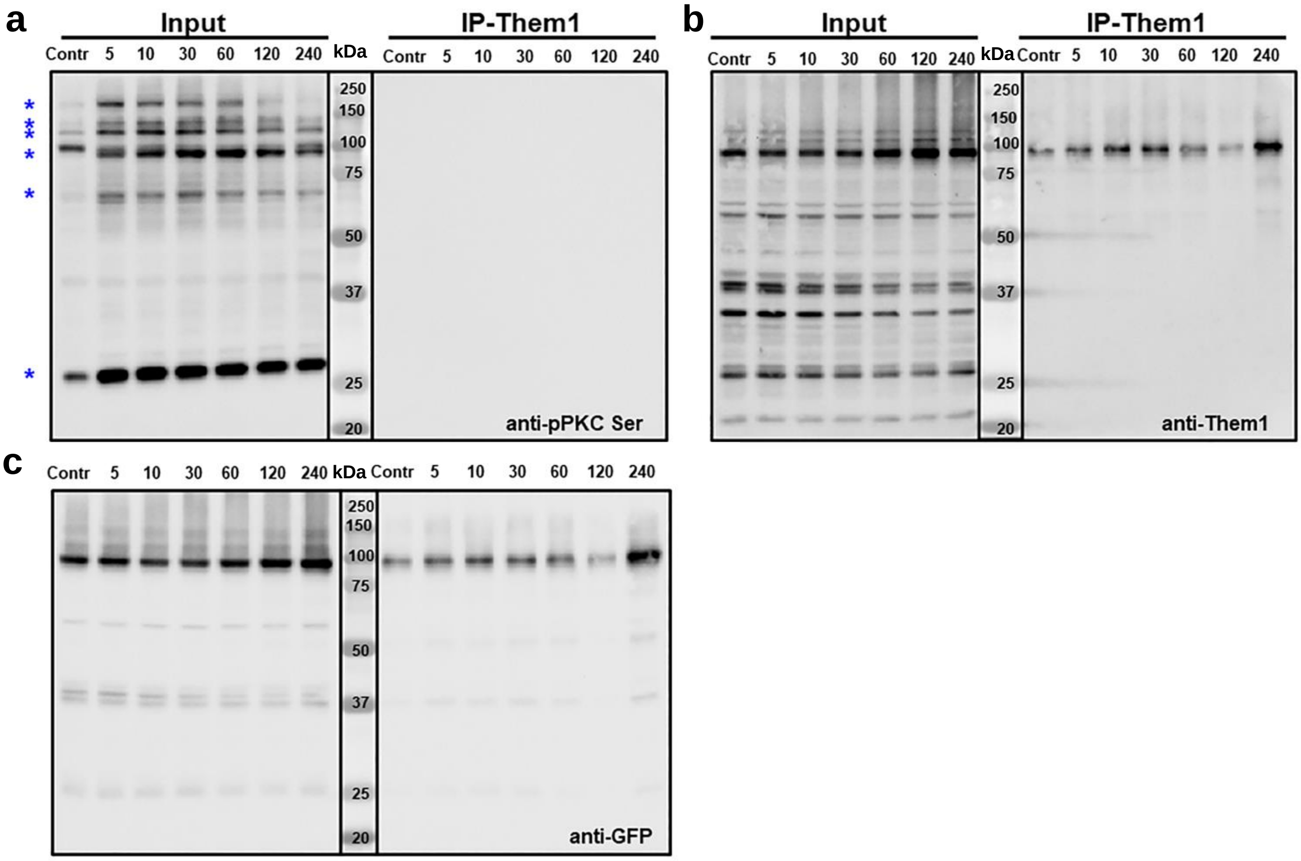

Supplementary Fig. 8 Phospho (P)-serine PKC substrate protein signature for immortalized brown adipose cells (iBAs) after stimulation with PMA. iBAs were transfected with Ad-Them1-EGFP and differentiated. Control (Contr) iBAs were untreated. Time points after PMA stimulation were from 5-240 minutes. Input is the cell lysate before immunoprecipitation (IP) and IP was done using an EGFP-trap. Antibodies used were anti-P-(serine) PKC substrate antibody (Cell Signaling Technology, #2261; anti GFP; anti-Them1 (see Supplementary Fig. 4). **a** Them1 (IP lanes) shows no P-serine peptides generated via PKC phosphorylation although the input lanes demonstrate that there are at least 6 candidate proteins in iBAs that are serine phosphorylated by PKC in a time-dependent manner (blue astericks). **b-c** Side-by-side gels/immunoblots were used to verify that Them1 and EGFP were present in samples subjected to the anti-P-serine PKC substrate antibody. Data are representative of 3 different immunoblots with the same results. Immunoblot data were not processed or modified from the original scans.

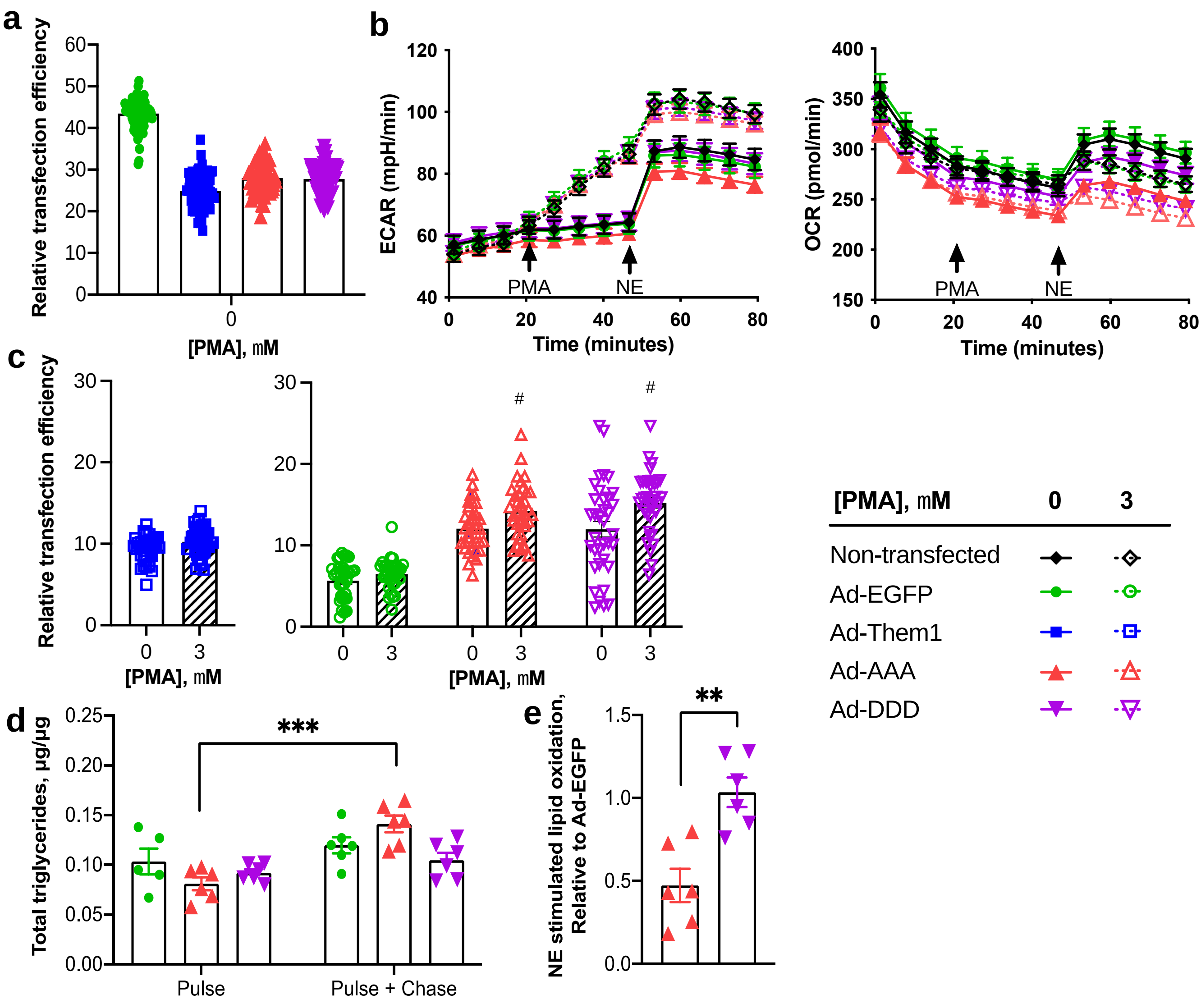

Supplementary Fig. 9 Pulse chase studies support the role of Them1-containing puncta in regulating fatty acid oxidation in immortalized brown adipose cells (iBAs). **a-c** iBAs were transduced with Ad-Them1-EGFP, Ad-AAA-EGFP, Ad-DDD-EGFP or Ad-EGFP (control), or not transduced (non-transfected control) and treated with 0 or 3 µM phorbol 12-myristate 13-acetate (PMA) 30 min before norepinephrine (NE) stimulation. **a,** Relative transfection efficiencies to support data in Fig. 4c. **b,** Extracellular acidification rate (ECAR) and oxygen consumption rate (OCR) to support data in Figure 5e,f. **c**, Relative transfection efficiencies to support data in Fig. 4d,e,f. Bars show mean ± SE of combined data for *n* = 3 independent experiments with technical replicates added to each bar. # *P*<0.05, 0 µM PMA vs 3 µM PMA (2-way ANOVA with Sidak's multiple comparison test). **d-e** Transfected iBAs were subjected to a pulse (9,10-^{3}H-oleate and 0.5 mM oleate) followed by a chase (0.5 mM oleate and 5 µM NE). **d**) Data show total intracellular triglyceride accumulation and **e**) data show lipid oxidation after norepinephrine (NE) stimulation, which was determined by utilization of triglycerides during the chase period and expressed relative to Ad-EGFP. Bars show mean ± SE from one experiment that is representative of *n*=2 independent experiments with individual technical replicates added to each bar. ****P*<0.001 Ad-AAA-EGFP pulse compared to pulse + chase (2-way ANOVA with Sidak's multiple comparison test); ***P*<0.01 Ad-AAA-EGFP compared to Ad-DDD-EGFP (Student's t-test).

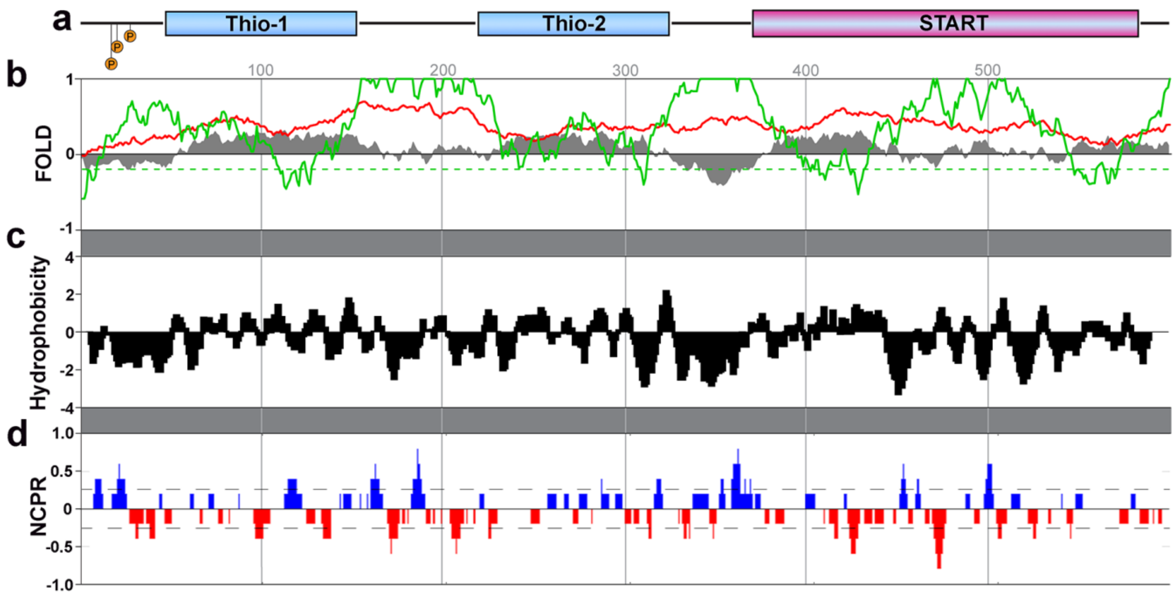

Supplementary Fig. 10 Bioinformatics to support the analysis in Fig. 6. a Schematic diagram of the Them1 amino acid sequence aligned with the amino acid number from the N- to C-terminus. Thio indicates the position of thioesterase domains 1 and 2 and START indicates the position of the START domain. **b** Results from the FOLD server, which predicts regions of intrinsic disorder by PLAAC (green) and the Prion Aggregation Prediction Algorithm (PAPA-red). The FOLD index is in grey. This analysis confirms the PLD server analysis (Fig. 6c) that Them1 has no prion-like regions in the sequence. **c** Hydrophobicity analysis shows that the Them1 N-terminal sequence contains a high proportion of charged residues with a patch of basic residues followed by a patch of acidic residues. These patches could potentially engage in non-covalent crosslinking with other proteins or itself to drive a phase transition. **d** NCPR shows the net charge per residue. This information can help to understand the loss of solubility of Them1, particularly in disordered regions. Dashed lines indicate the significance threshold.
