# Supplementary material for: Thioesterase superfamily member 1 undergoes stimulus-coupled conformational reorganization to regulate metabolism in mice
Source: Nat Commun. 2021 Jun 9;12:3493. doi: 10.1038/s41467-021-23595-x (PMC8190112; doi:10.1038/s41467-021-23595-x)
Supplement: Supplementary file 3 — Reporting Summary [file 41467_2021_23595_MOESM3_ESM.pdf]

Corresponding author(s): Susan J. Hagen
Last updated by author(s): Jan 14, 2021

Reporting Summary

Nature Research wishes to improve the reproducibility of the work that we publish. This form provides structure for consistency and transparency in reporting. For further information on Nature Research policies, see our Editorial Policies and the Editorial Policy Checklist.

Statistics

For all statistical analyses, confirm that the following items are present in the figure legend, table legend, main text, or Methods section.

n/a	Confirmed	
☐	☒	The exact sample size (*n*) for each experimental group/condition, given as a discrete number and unit of measurement
☐	☒	A statement on whether measurements were taken from distinct samples or whether the same sample was measured repeatedly
☐	☒	The statistical test(s) used AND whether they are one- or two-sided<br>*Only common tests should be described solely by name; describe more complex techniques in the Methods section.*
☐	☒	A description of all covariates tested
☒	☐	A description of any assumptions or corrections, such as tests of normality and adjustment for multiple comparisons
☐	☒	A full description of the statistical parameters including central tendency (e.g. means) or other basic estimates (e.g. regression coefficient) AND variation (e.g. standard deviation) or associated estimates of uncertainty (e.g. confidence intervals)
☐	☒	For null hypothesis testing, the test statistic (e.g. *F*, *t*, *r*) with confidence intervals, effect sizes, degrees of freedom and *P* value noted<br>*Give P values as exact values whenever suitable.*
☒	☐	For Bayesian analysis, information on the choice of priors and Markov chain Monte Carlo settings
☒	☐	For hierarchical and complex designs, identification of the appropriate level for tests and full reporting of outcomes
☒	☐	Estimates of effect sizes (e.g. Cohen's *d*, Pearson's *r*), indicating how they were calculated

Our web collection on statistics for biologists contains articles on many of the points above.

Software and code

Policy information about availability of computer code

Data collection	Zen 2.3 SP1 for confocal image collection; ChemiDoc for Western-blot; Seahorse Wave controller 2.4 for oxygen consumption rates data collection; JEOL 1400 electron microscope system with Gatan Digital Micrograph 3.32.2403.0 software for EM image collection; SpectraMax 190 microplate reader
Data analysis	Volocity 6.5; Imaris x64 9.6.0; Seahorse Wave controller 2.4; SigmaPlot14 for statistics; SoftMax Pro 6.5.1 ; Proteome Discoverer v2.4

For manuscripts utilizing custom algorithms or software that are central to the research but not yet described in published literature, software must be made available to editors and reviewers. We strongly encourage code deposition in a community repository (e.g. GitHub). See the Nature Research guidelines for submitting code & software for further information.

Data

Policy information about availability of data

All manuscripts must include a data availability statement. This statement should provide the following information, where applicable:
- Accession codes, unique identifiers, or web links for publicly available datasets
- A list of figures that have associated raw data
- A description of any restrictions on data availability

The authors declare that the main data supporting the findings of this study are available within the article and its Supplementary Information files. Adenoviral vectors or plasmid constructs are available upon reasonable request to the corresponding authors.

Field-specific reporting

Please select the one below that is the best fit for your research. If you are not sure, read the appropriate sections before making your selection.

☒ Life sciences ☐ Behavioural & social sciences ☐ Ecological, evolutionary & environmental sciences

For a reference copy of the document with all sections, see nature.com/documents/nr-reporting-summary-flat.pdf

Life sciences study design

All studies must disclose on these points even when the disclosure is negative.

Sample size	No sample-size calculation was performed. We chose the sample-size larger than 3 for all experiments. For each study, the sample size needed to provide robust power to determine significant differences. If it did not, additional experiments were performed.
Data exclusions	When doing the animal experiments, the data from one control mouse and one injected mouse was a complete outlier looking rather like the opposite treatment. On the day of experiments, we had a lot of people collecting various aspects of the study and assume in this case the tissues were put into the wrong vial for histology. We thus excluded these two tissues and had an n=2 rather than n=3 for those treatments.
Replication	All the experiments with cell culture were repeated three times or more. All of the replications showed the same result.
Randomization	We picked random areas / cells on the microscopy slides to capture images.
Blinding	Yes

Reporting for specific materials, systems and methods

We require information from authors about some types of materials, experimental systems and methods used in many studies. Here, indicate whether each material, system or method listed is relevant to your study. If you are not sure if a list item applies to your research, read the appropriate section before selecting a response.

Materials & experimental systems

n/a	Involved in the study	
☐	☒	Antibodies
☐	☒	Eukaryotic cell lines
☒	☐	Palaeontology and archaeology
☐	☒	Animals and other organisms
☒	☐	Human research participants
☒	☐	Clinical data
☒	☐	Dual use research of concern

Methods

n/a	Involved in the study	
☒	☐	ChIP-seq
☒	☐	Flow cytometry
☒	☐	MRI-based neuroimaging

Antibodies

Antibodies used

Anti-Tomm20: Santa Cruz Biotech, clone: FL-145, Cat: sc-11415, Lot: D0616;
Anti-Plin1: Fitzgerald, Cat: 20R-PP004, Lot: P14062427;
Anti-GFP: Novus Biologicals, Cat: NB100-1678, Lot: 30178;
Anti-actin: Abcam, clone: C4, Cat: Ab3280, Lot: 899845;
Anti-Them1: Covance Research Products, #379
Anti-Phospho-(Ser) PKC Substrate: Cell Signaling Technology, Inc., Cat: 2261S, Lot: 01/2020;
Anti-Rabbit-HRP: Jackson ImmunoResearch Laboratories, Inc., Cat: 711-035-152, Lot: 142507;
Anti-Mouse-HRP: Jackson ImmunoResearch Laboratories, Inc., Cat: 715-035-150
Cy3-conjugated Anti-Guinea Pig: Jackson ImmunoResearch Laboratories, Inc., Cat: 706-165-148, Lot: 115370

Validation

Anti-Tomm20: a rabbit polyclonal IgG, cross reactivity: mouse, rat, human; applications: IP, WB, IF, IHC, ELISA
Anti-Plin1: Guinea Pig polyclonal Perilipin antibody, using duplicated N-terminus of perilipin as the immunogen; cross reactivity: human, mouse, rat; applications: IHC-F, IHC-P, WB
Anti-GFP: Goat polyclonal antibody against a fusion protein corresponding to the full length GFP (246aa) derived from the jellyfish Aequorea victoria; applications: WB, ELISA, ICC/IF, IHC, IHC-Fr, IHC-P, KD, KO; validation: knockdown and knockout validation.
Anti-actin: Mouse monoclonal [C4] against to Chicken Actin aa 50-70; application: ICC/IF, WB; Reacts with: Chicken, Saccharomyces cerevisiae, Tetrahymena, Caenorhabditis elegans, Mammals, Dictyostelium discoideum, Physarum polycephalum, Vertebrata
Anti-Them1: Rabbit anti-mouse Them1 polyclonal antibody was produced to against the peptide (PQKLPWIRPQPGEGERRY) designed to match the amino acid sequence 333–550 of Them1.
Anti-Phospho-(Ser) PKC Substrate: Rabbit polyclonal antibody; cross reactivity: all species; applications: IP, WB.
Anti-Rabbit-HRP: Target: Rabbit IgG(H+L)
Anti-Mouse-HRP: Target: Mouse IgG(H+L)

	Cy3-conjugated Anti-Guinea Pig: Target: Guinea Pig IgG(H+L)

Eukaryotic cell lines

Policy information about cell lines

Cell line source(s)	The iBAs cells were obtained from Dr. Bruce Spiegelman lab
Authentication	PCR analysis of genes specific to BAT before and after norepinephrine stimulation identifies these cells as brown adipocytes.
Mycoplasma contamination	The cell line has been confirmed with no mycoplasma contamination by PCR.
Commonly misidentified lines (See ICLAC register)	No commonly misidentified cell lines were used in the study.

Animals and other organisms

Policy information about studies involving animals; ARRIVE guidelines recommended for reporting animal research

Laboratory animals	7-weeks old male C57BL/6J mice
Wild animals	This study did not involve wild animals.
Field-collected samples	This study did not involve samples collected in the field.
Ethics oversight	Animal use and euthanasia protocols were approved by the Institutional Animal Care and Use Committee at Weill Cornell Medical College.

Note that full information on the approval of the study protocol must also be provided in the manuscript.
